# Supplementary figures and images for: Clinicopathological Features and Prognosis of Lung Adenocarcinoma Presenting as Ground-Glass Opacity: A Single-Center Experience
Source: Cancers (Basel). 2025 Sep 16;17(18):3016. doi: 10.3390/cancers17183016 (PMC12468804; doi:10.3390/cancers17183016)

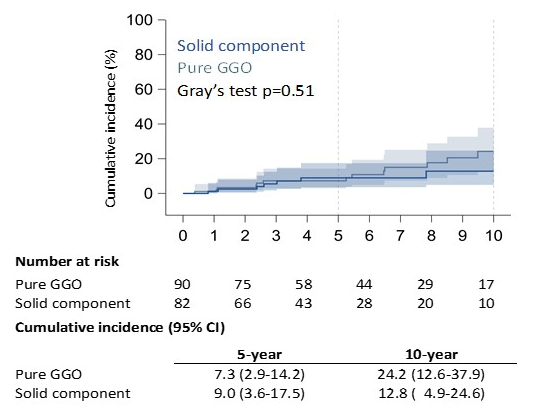

Supplement: Supplementary file 1 [file cancers-17-03016-s001.zip › Supplementary Figure S1.jpg]

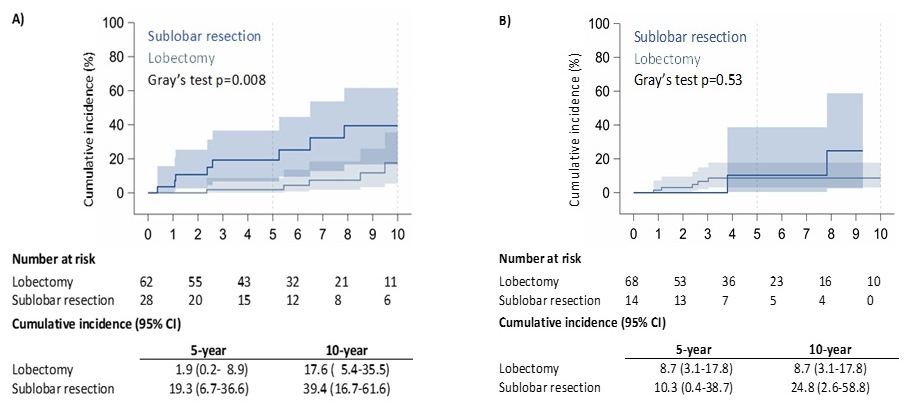

Supplement: Supplementary file 1 [file cancers-17-03016-s001.zip › supplementary Figure S2.jpg]
